# Nurse Interns' Perception of Clinical Preparation and Readiness for Clinical Internship Experiences

**DOI:** 10.1155/2024/6682600

**Published:** 2024-03-15

**Authors:** Noura Almadani, Reda M. Hables, Jalal Alharbi, Majed Alamri, Mukhlid Alshammari

**Affiliations:** ^1^Community and Psychiatric Mental Health Nursing Department, College of Nursing, Princess Nourah bint Abdulrahman University, Riyadh 84428, Saudi Arabia; ^2^Nursing Department, College of Applied Medical Sciences, University of Hafr Al Batin, Hafr Al Batin 39524, Saudi Arabia

## Abstract

**Background:**

As nursing interns enter challenging clinical settings, evaluating their preparation and readiness is vital for adaptation success. Sufficient real-world experience and patient care are crucial preparation components that enable effective practice and higher competencies.

**Aim:**

This study aimed to assess nurse interns' perception of clinical preparation and readiness for clinical internship experiences.

**Methods:**

A self-administered questionnaire was distributed, comprising three sections on demographics, clinical preparation requirements, and the Casey–Fink Readiness for Practice Survey. Descriptive statistics as the mean and standard deviation, numbers and percentages, linear regression model, and Pearson correlation coefficient were used for reporting normal distribution, categorical variables, and relationship between a scalar response and one or more explanatory variables and to calculate statistics between two continuous variables.

**Results:**

The participants were 130 nurse interns who were involved in an internship between 2016 and 2020, 50% of the nurse interns. They had a moderate level of clinical preparation, and 28.5% of them exhibited a low level. In addition, 53.8% were found to be moderately ready for practice, while 22.3% had a low level of readiness

**Conclusion:**

The observed significant positive correlation between perceived preparation and readiness underscores the pivotal role of clinical preparation in influencing practice readiness. *Implications*. These findings emphasize the importance of targeted interventions aimed at enhancing clinical preparation to directly bolster overall readiness for professional practice among nurse interns.

## 1. Introduction

Adequate clinical preparation and readiness are vital for nursing interns entering challenging hospital environments. However, studies indicate inconsistencies in nursing students' perceived preparation, highlighting a crucial need to evaluate preparation programs [1]. As rising patient acuities increase demands on novice nurses, sufficient clinical skills and competencies determine adaptation success during role transitions from student to practicing nurse [2]. The intricacy of modern healthcare environments means nursing graduates must possess clinical reasoning, critical thinking, organizational skills, and communication capacities [3].

Given that essential skills are primarily developed through clinical placements, it is concerning that studies report discrepancies between students' perceived readiness and actual workplace readiness [4]. Determining alignment requires simultaneous investigation into multiple preparation elements including knowledge, nursing skills, critical thinking abilities, ethical comportment, and professional identity. Understanding where potential gaps exist allows educational initiatives targeting identified deficiencies. [5].

Adequate clinical preparation is vital for nursing students transitioning into demanding healthcare environments. However, research indicates inconsistencies in students' perceived readiness, highlighting a crucial need to evaluate preparation programs [6]. Modern healthcare requires graduates have clinical reasoning, critical thinking, organizational skills, and communication capacities [5].

Clear clinical instruction assists nursing students through the transition period from student to registered, professional nurse [7]. To be competent, the nurse intern must have scientific/theoretical knowledge, particular technical psychomotor abilities, communication skills, professional values, and ethical behavior to provide care for patients in real situations [8].

Education plays a major role in the advancement of nursing discipline. Several programs, both undergraduate and postgraduate, have been developed to train nursing student. An educational program that can apply these characteristics is able to cover a large number of skills and traits that are essential in the professional environment [9].

The five-year Bachelor of Science in Nursing (BSN) program at most Saudi Arabia universities contains a mix of science and arts courses which advance nursing clinical practice and competency [10]. The program includes four years of theoretical education, and in the fifth year, a nursing internship year, which provides direct patient care experience in a healthcare setting [11].

An internship is designed to prepare the nurse intern for the different skills needed to be successful in the profession [12]. The one-year internships in Saudi Arabia rotate the nursing student through different clinical settings, including medical-surgical for pediatrics and adults, critical care in intensive care units, the emergency room, the operating room, triage area, dialysis units, labor and delivery units, and outpatient clinic [13].

Our study aimed to assess nurse interns' perceptions of their clinical preparation and readiness and examine relationships between perceived preparation and perceived practice readiness. The goal is to gain insight into the alignment between nurse interns' perceived capacities and workplace demands in order to guide educational approaches that optimize clinical preparation. Enhancing readiness aims to ease the role transition from nursing student to practicing nurse, facilitating better adaptation and competency as the next generation enters the healthcare workforce.

### 1.1. Research Questions

1. Whet is nurse interns' perception of clinical preparation and readiness for clinical internship experiences?
2. Are there significant correlations between nurse interns' perceived level of clinical preparation and their self-reported readiness for nursing practice during the internship?

## 2. Methods

A cross-sectional research design was utilized. This study was carried out at the College of Applied Medical Sciences, Hafr Al Batin University, Saudi Arabia.

### 2.1. Sampling

Participants for the study were recruited by homogeneous purposive sampling. The participants were 130 nurse interns. The study participants joined to this study according to the following inclusion criteria: the participants willing to participate in the study. The nurse interns enrolled in their internship year, which is the culmination of a graduate program consisting of a foundation year and a three-year baccalaureate program. The program also includes a mandatory 12-month internship, allowing nursing interns to gain practical experience in government hospitals.

There were 130 nurses involved in an internship between 2016 and 2020 (Table 1).

### 2.2. Data Collection

A self-administered questionnaire was utilized to collect data, covering aspects related to demographic characteristics, clinical preparation, and readiness for practice.

The questionnaire was translated into Arabic to ensure clarity and accessibility for participants.

Content validity was assured by a jury of 5 experts in the field of nursing management and nursing education and community health nursing.

For the first three groups (2016–2019), which represent nurse interns from the first three graduated years (2016–2019), data collection involved in-person interviews. The researcher conducted individual interviews with each nurse intern included in the study. During the interviews, participants responded to the questionnaire items, and the researcher recorded their responses to encourage interns and prevent hesitant to disclose information.

For the last year (2019-2020), researchers opted for an online data collection approach. The tools were distributed to nurse interns electronically through an online platform. The questionnaire was distributed via an official email specifically designated for nurse interns, ensuring confidentiality and targeted outreach. The response rate of 100% was calculated by dividing the number of valid responses by the total number of responses requested during interview and the number of emails replies compared to the number of emails sent.

### 2.3. Instruments

#### 2.3.1. Part I: Characteristics of Nurse Interns

The characteristics of nurse interns were age, marital status, type of family, graduated year, last grade point average (GPA), what orientation program they attended, and whether it was effective.

Content validity was assured by a jury of 5 experts in the field of nursing management and nursing education and community health nursing.

#### 2.3.2. Part II: Clinical Preparation Requirement Questionnaires

This tool was adapted from Hickey [14]. The tool comprises six components and a total of 45 items. These items are designed to evaluate the six critical components of necessary clinical preparation, namely, nursing process steps, use of resources, psychomotor skills, teaching and information imparted, communication, and administrative aspects. Respondents rated each item on a scale ranging from (1) “not important” to (4) “essential.” The overall scale, with a possible range from 45 to 180, allowed for categorization based on importance; scores below 60 were considered of low importance, scores between 60 and 120 denoted moderate importance, and scores exceeding 120 indicated high importance.

Reliability testing was performed using Cronbach's alpha, resulting in a value of 0.856.

Content validity was assured by a jury of 5 experts in the field of nursing management and nursing education and community health nursing.

#### 2.3.3. Part III: Casey–Fink Readiness for Practice Survey

This scale was adapted from Casey et al. [15]. The selection of the tool is based on its utilization in previous research [16]. This tool was originally proposed by Kathy Casey and Regina Fink to measure newly licensed registered nurses' comfort with skills over time. This part comprises 20 items distributed across four domains of readiness during an internship. The domains include clinical problem solving (7 items), learning techniques (2 items), professional identity (5 items), and trials and tribulations (6 items). Participants rated each item on a scale ranging from (1) “strongly disagree” to (4) “strongly agree.” The total scores for this scale ranged from 20 to 80, with response categorization as follows: scores below 27 were considered of low importance, scores between 27 and 53 denoted moderate importance, and scores exceeding 53 indicated high importance.

Reliability testing was performed using Cronbach's alpha, resulting in a value of 0.839.

Content validity was assured by a jury of 5 experts in the field of nursing management and nursing education and community health nursing.

### 2.4. Pilot Study

A pilot study was conducted with 13 nurse interns, representing 10% of the study cohort, to assess the construction and clarity of the tool. This pilot study also aimed to determine the time required for each participant to complete the questionnaire. The tools proved to be clear, and no modification was needed.

### 2.5. Statistical Analysis

Data were analyzed via the Statistical Package for Social Science (SPSS) software, version 23 (SPSS Inc. Chicago, IL, USA). Descriptive statistics as the mean and standard deviation were used for reporting normally distributed numerical variables. Numbers and percentages were used to describe categorical variables. A linear regression model was used to assess the relationship between a scalar response and one or more explanatory variables. The Pearson correlation coefficient was used to calculate statistics between two continuous variables.

### 2.6. Ethical Considerations

Institutional Review Board (IRB) approval was obtained from the local authoritative body. Written informed consent was obtained before data were collected during interwire or via an official email. Each nurse intern was knowledgeable about the aim of the study before participation. They were informed that their participation in the study was elective, and they had the decision to refuse participation at any time of the study without penalties. All patients' data were confidential and used only for research purposes.

## 3. Results

Table 1 provides sociodemographic details of participating nurses. The mean age of the nurses was 26.09 (SD = 3.96) years, and all participants were female. The majority were unmarried, with two fifths (40%) holding a GPA of 3.75–4.49. In addition, all nurses attended orientation programs, with approximately half (49.2%) of them strongly agreeing on the effectiveness of their respective programs.

According to readiness for practice, Table 2 revealed that clinical problem-solving had the highest mean percentage at 70.5%, with a mean score of 19.745 ± 3.61. In contrast, the lowest-scoring domain was trials and tribulations with a mean score of 4.23 ± 1.01, with mean percentage 52.9%.

Regarding to the importance of clinical preparation, Table 3 detected that the mean score for the steps of the nursing process was 36.82 ± 9.76, representing the lowest mean percentage at 41.8%. In addition, the mean score for the use of resources was 3.86 ± 0.98, and for teaching and information giving, it was 19.56 ± 6.60. On the other hand, communication skills had a mean score of 13.51 ± 4.49, with the highest mean percentage of 84.4%. Administrative skills had a mean score of 10.11 ± 1.95, accounting for 50.6%, as mentioned in Table 4.

Table 4 displays the results of the multiple linear regression model examining the perception related to clinical preparation requirements. The model shows a slight but significant fit, as indicated by the F-test value of 11.965 with a *p* value of 0.002. Approximately, 51% of the variation in clinical preparation requirements is explained by this model, as reflected by the *R*^2^ value of 0.511.

Moreover, the analysis suggests that an increase in age by one unit is associated with a corresponding increase in perception related to preparation requirements by 0.169. Similarly, an increase in GPA by one unit is linked to a higher perception, showing an increment of 0.355. Notably, nurses who graduated in 2019 and 2020 demonstrated a higher perception compared to those who graduated in 2016-2017.

Table 5 illustrates that the multiple linear regression model for the Casey–Fink Readiness for Practice Survey indicates a statistically significant fit, as evidenced by the F-test value of 9.863 with a *p* value of 0.004. This model accounts for approximately 54% of the variation in the Casey–Fink Readiness for Practice Survey, as denoted by the *R*^2^ value of 0.540.

The findings further reveal that an increase in GPA by one unit is associated with a corresponding increase in study subjects' readiness by 0.398. In addition, unmarried interns exhibit a higher level of readiness compared to married interns, with a difference of 0.171. Moreover, an increase in the perception of the orientation program by one unit is linked to an increased readiness by 0.189, see more in Table 6.

Table 6 shows that there was a highly significant correlation between the readiness for practice and clinical preparation requirements at *p* value <0.01.

**Figure 1:**
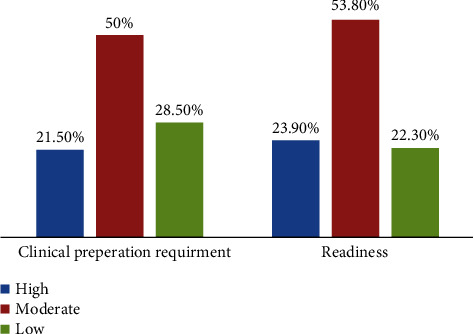
Distribution of nurse interns-related clinical preparation and readiness for practice (*n* = 130).

**Figure 2:**
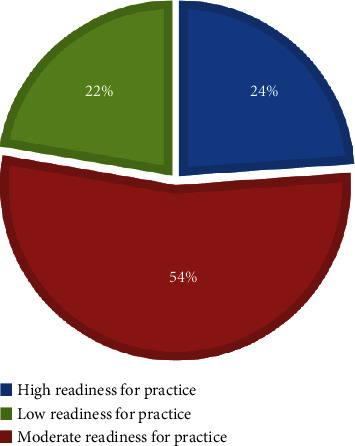
Distribution of nurse interns-related readiness for practice (*n* = 130).

Figures 1 and 2 show that 50% of nurse interns had a moderate level of clinical preparation, and 28.5% of them exhibited a low level. In addition, 53.8% were found to be moderately ready for practice, and 23.90% had a highly ready for practices, while 22.3% had a low level of readiness.

## 4. Discussion

Navigating the shift from being a nursing student to a nurse intern is often difficult. Assessing how equipped students are for workplace demands can ease the transition process. Intern readiness to deliver secure, skilled care is an urgent issue because of rising workloads and elaborate healthcare frameworks. Ramifications of unsatisfactory preparation during this move can be examined both individually and organizationally. The effect of inadequate preparedness during transition can be viewed from the personal and from the system level.

Regarding readiness to practices, this study found that the highest mean percentage (70.5% )of new nurses feltmost ready to practices regardingthe domain of clinical problem solving. While, 52.9% of themthey felt that the least read to practices regardingthe domain of trials and tribulations.

These results are incongruent with [17], who reported that final-year undergraduate nursing students in a school in the Republic of Ireland are concerned about their readiness for practice. While Aswad et al. [18] revealed that only one quarter of study participants were highly ready.

Regarding specific clinical preparation skills, the current study revealed that communication skills were the strongest area reported with a mean percentage of 84.4%. However, applying the full nursing process and knowing how to utilize informational resources were relative reported weakness with a mean percentage of 41.8%. These results cohort with the study by Abdelkader et al. [19] who found that the highest scores for educational preparation requirements was in teaching and information giving, psychomotor skills, and communications skills (79.70%, 78.82%, and 76.92%, respectively). Furthermore, another study [20] reported that the majority of nurse interns (a mean percent of 50.6%) needed training in managerial skills. Nicholls [21] stated that both psychomotor and nursing process skills were extremely important requirements for clinical preparation assignments.

The study findings revealed that there was a highly significant correlation between the readiness level and perceived preparation requirements also underscores the importance of alignment. Nursing schools must adequately equip students for the real demands of clinical practice. Meanwhile, healthcare institutions must recognize the limitations of novice nurses and support their competent acclimation to the provider role. These results, similar to the study by Babaei et al. [22], found that there was a positive and significant correlation between nurses' attitudes and readiness. In addition, Ahmadi et al. [23] recommended that the clinical preparation of nursing students during the internship program enhances clinical readiness in internships.

The regression models provide further insight into factors impacting nurse interns' preparation and readiness for practice. This study revealed a slight but significant fit relationship, as indicated by the F-test value of 11.965 with a *p* value of 0.002, which indicate that older nurse age and higher nursing GPA were associated with greater perceived preparation requirements and overall readiness levels. Similarly, an increase in GPA by one unit is linked to a higher perception, showing an increment of 0.355. These results go online with the study by [24] who detected that age (*β* = 0.029, SE = 0.012, and *p*=0.021) and employment orientation duration (*β* = −0.007, SE = 0.003, and *p*=0.018) were statistically significant factors influencing role transition experience among newly joined nurses. In addition, Alshammari et al. [25] mentioned that the GPA of nurse interns had a positive correlation on perception and adaptation during the training program, and Kaur [26] reported the importance of developing a positive interpersonal relationship with patients in nursing practice.

Furthermore, nurse interns more recently in 2019-2020 felt better prepared than those from 2016 to 2017. Notably, nurses who graduated in 2019 and 2020 demonstrated a higher perception compared to those who graduated in 2016-2017. Marital status and orientation program quality also appeared to influence readiness. Likewise, a study by Ahmed et al. [27] revealed that the marital status had no relation to nurse intern performance.

Also, Harrison et al. [28] found that an effective orientation program had a positive effect on increasing the standard of care. On the other hand, Sharma et al. [29] stated that there was no association between nurse intern clinical competence and their satisfaction with an internship program.

With careful attention to rounding out nursing curricula, standardizing orientation programs, and bridging the transition into practice, new nurses' high levels of clinical knowledge can be translated into comprehensive readiness. This will empower them to deliver excellent care while building confidence in their evolving skills. At this context, Babamohamadi et al. [30] mentioned that the knowledge level of nurses' interns improves their readiness to the clinical situations. Also, Permana et al. [31] demonstrated a statistically significant correlation between students' caring conduct and clinical preparation.

### 4.1. Implication for Practice

Providing ongoing professional development opportunities for nurse interns can contribute to the continuous improvement of their skills and readiness. Encouraging collaboration between nursing education programs and healthcare institutions can foster a seamless transition from education to practice. Establishing mentorship programs can provide invaluable support for nurse interns.

### 4.2. Strength and Limitation

The strength of this study was the selection of topics related to perceived preparation and readiness of nurse internees. One of the limitations of this study was related purposive sampling technique. Further studies with more sample size using the randomized sampling technique needs to be conducted for large size of participants and different colleges in Saudia Arabia.

## 5. Conclusion

In conclusion, the study reveals that a substantial proportion of nurse interns, precisely half, displayed a moderate level of clinical preparation, while over one-quarter exhibited a low level. In addition, more than half of the interns were deemed moderately ready for practice, with over one-fifth categorized as having a low level of readiness. The observed significant positive correlation between perceived preparation and readiness underscores the pivotal role of clinical preparation in influencing practice readiness. These findings emphasize the importance of targeted interventions aimed at enhancing clinical preparation to directly bolster overall readiness for professional practice among nurse interns.

## Figures and Tables

**Table 1:** Characteristics of studied nurse interns (*n* = 130).

Items	*N*	%
Age (year)		
20–<25 years	35	26.9
25–30 years	95	73.1
Mean SD	26.09 ± 3.96	
Gender		
Female	130	100
Marital status		
Married	15	11.5
Unmarried	115	88.5
Last GPA		
<2	9	6.9
2–2.74	17	13.1
2.75–3.74	33	25.4
3.75–4.49	52	40
>4.49	19	14.6
Type of family		
Nuclear	50	38.5
Extended	80	61.5
Graduation year		
2016-2017	15	11.5
2017-2018	32	24.6
2018-2019	20	15.4
2019-2020	63	48.5
Attended orientation program		
Yes	130	100
Perception related effective of program of orientation		
Strongly agree	64	49.2
Agree	40	30.8
Disagree	20	15.4
Strongly disagree	6	4.6

**Table 2:** Readiness for practice among studied intern nurses (*n* = 130).

Items	Mean score	Mean percent
Clinical problem solving	19.745 ± 3.61	70.5
Professional identity	11.03 ± 2.50	55.2
Learning techniques	12.97 ± 4.02	54.04
Trials and tribulations	4.23 ± 1.01	52.9

**Table 3:** Clinical preparation requirements for intern nurses (*n* = 130).

Items	Mean SD	Mean percent
Communication skills	13.51 ± 4.49	84.4
Teaching and information giving	19.56 ± 6.60	69.9
Psychomotor skills	12.88 ± 4.07	64.4
Administrative skills	10.11 ± 1.95	50.6
Uses of resources	3.86 ± 0.98	48.2
Step of nursing process	36.82 ± 9.76	41.8

**Table 4:** Best fitting multiple linear regression model for perception related to clinical preparation requirements.

	Unstandardized coefficient		Standardized coefficient	*T* test	*p* value
	*B*	Std. error			
Age	0.169	0.209	0.189	5.017	0.011
Last GPA	0.355	0.321	0.246	7.894	0.007
Graduated year	0.197	0.187	0.199	6.102	0.009

**Table 5:** Best fitting multiple linear regressions model for Casey–Fink Readiness for Practice Survey.

	Unstandardized coefficient		Standardized coefficient	T- test	*p* value
	*B*	Std. error			
Last GPA	0.398	0.257	0.157	4.135	0.010
Marital status	0.171	0.199	0.142	3.996	0.022
Perception related effective of orientation program	0.189	0.196	0.172	3.746	0.024

**Table 6:** Correlation between clinical preparation requirements and Casey–Fink Readiness for Practice Survey.

	Casey–Fink Readiness for Practice Survey
Clinical preparation requirements	*r*. 0.598
	*p* 0.001

## Data Availability

The data used to support the findings of this study are available from the corresponding author upon request.

## References

[1] Chao Y. C., Hu S. H., Chiu H. Y., Huang P. H., Tsai H. T., Chuang Y. H. (2021). The effects of an immersive 3d interactive video program on improving student nurses’ nursing skill competence: a randomized controlled trial study. *Nurse Education Today*.

[2] Sterner A., Ramstrand N., Palmér L., Hagiwara M. A. (2021). A study of factors that predict novice nurses’ perceived ability to provide care in acute situations. *Nursing Open*.

[3] Assarroudi A., Heshmati Nabavi F., Ebadi A., Esmaily H. (2017). Professional rescuers’ experiences of motivation for cardiopulmonary resuscitation: a qualitative study. *Nursing and Health Sciences*.

[4] Missen K., McKenna L., Beauchamp A. (2016). Registered nurses’ perceptions of new nursing graduates’ clinical competence: a systematic integrative review. *Nursing and Health Sciences*.

[5] Anthony J. P. (2022). Nurse clinical reasoning: comparing graduate nurse perceptions with faculty assessments across three nursing preparation program types.

[6] Holch U. P. (2022). Critical thinking skills in nurses: perception of graduate nurses.

[7] Tumala B. (2021). Predictors of nursing interns’ standard precautions compliance during internship training in four teaching hospitals in Saudi Arabia. *International Journal of Nursing Practice*.

[8] Yang C., Zhu Y., Xia B., Li W., Zhang J. (2020). The effect of structured empathy education on empathy competency of undergraduate nursing interns: a quasi-experimental study. *Nurse Education Today*.

[9] Tener R., Winstead M., Smaglik E. (2001). Experiential learning from internships in construction engineering. *ASEE Annual Conference and Exposition: Peppers, Papers, Pueblos and Professors*.

[10] Alabdulaziz H., Alquwez N., Cruz J., Tumala R., Albougami A., Albloushi M. (2021). The Compassion Competence Scale Arabic version: a validation study among student nurses and interns in Saudi Arabia. *International Journal of Nursing Practice*.

[11] Al Najjar H., Bano N. (2020). Experiences of nursing interns with the application of knowledge and skills in drug administration: a qualitative study. *Saudi Journal for Health Sciences*.

[12] Ayaz-Aklaya S., Yaman-Sözbir Ş., Bayrak-Kahraman B. (2020). The effect of nursing internship program on burnout and professional commitment. *Nurse Education Today*.

[13] González-García M. P., Lana A., Zurrón-Madera P., Valcárcel-Álvarez Y., Fernández-Feito A. (2020). Nursing students’ experiences of clinical practices in emergency and intensive care units. *International Journal of Environmental Research and Public Health*.

[14] Hickey M. (2010). Baccalaureate nursing graduates’ perceptions of their clinical instructional experiences and preparation for practice. *Journal of Professional Nursing*.

[15] Casey K., Fink R., Jaynes C., Campbell L., Cook P., Wilson V. (2011). Readiness for practice: the senior practicum experience. *Journal of Nursing Education*.

[16] Cline D., La Frentz K., Fellman B., Brassil K. (2017). Longitudinal outcomes of an institutionally developed nurse residency program. *The Journal of Nursing Administration*.

[17] Leufer T., Cleary-Holdforth J. (2020). Senior nursing students’ perceptions of their readiness for practice prior to final year internship: part 2—a qualitative perspective. *Dimensions of Critical Care Nursing*.

[18] Aswad S. M., Al-Dabbagh S. A., Agha S. Y., Alsheikh G. Y. (2020). Assessment of readiness of newly graduated health professionals to communicate with patients in Duhok, Kurdistan Region, Iraq. *Journal of Health Medicine and Nursing*.

[19] Abdelkader A., Abundo R., Baratas G. (2021). Perceived level of preparation for nursing internship. *International Journal of Nursing Education*.

[20] Elhanafy E., El Hessewi G. (2020). Implementing management strategies to develop nurse’s intern managerial skills. *Journal of Integrated Health Science*.

[21] Nicholls D., Sweet L., Muller A., Hyett J. (2018). A model to teach concomitant patient communication during psychomotor skill development. *Nurse Education Today*.

[22] Babaie L., Haririan H., Rahmani A. (2022). Iranian nurses’ attitudes and readiness for nurse prescribing: a cross-sectional study. *Journal of Holistic Nursing And Midwifery*.

[23] Ahmadi S., Abdi A., Nazarianpirdosti M., Rajati F., Rahmati M., Abdi A. (2020). Challenges of clinical nursing training through internship approach: a qualitative study. *Journal of Multidisciplinary Healthcare*.

[24] Al-Rawajfah O. M., AlBashayreh A., Sabei S. D. A., Al-Maqbali M., Yahyaei A. A. (2023). Role transition from education to practice and its impact on the career futures of Omani nurses. *Nurse Education in Practice*.

[25] Alshammari F., Pangket P., Llego J., Pasay-an E., Gonzales F. (2020). Adaptation of nurse interns in acute hospital care practice and their demographic features: a correlation study. *International Journal of Innovation, Creativity and Change*.

[26] Kaur B. (2020). Interpersonal communications in nursing practice – key to quality health care. *Archives of Nursing Practical Care*.

[27] Ahmed R., Abdel-Azeem A. (2022). Problems facing intern nursing students and its relation with their perceived stress. *Egyptian Journal of Health Care*.

[28] Harrison H., Birks M., Franklin R., Mills J. (2019). Fostering graduate nurse practice readiness in context. *Collegian*.

[29] Sharma S. K., Kalal N., Rani R. (2021). Clinical practice readiness of nursing graduates. *Clinics in Mother and Child Health*.

[30] Babamohamadi H., Aghaei N., Asgari M. R., Dehghan-Nayeri N. (2023). Strategies used by Iranian nursing students for adjusting to internship: a qualitative study. *BMC Medical Education*.

[31] Permana B., Yusuf A., Setiawan H., Putri T. A. R. K. (2023). Nursing students’ caring behavior towards clinical learning readiness. *Jurnal Keperawatan Komprehensif (Comprehensive Nursing Journal)*.

